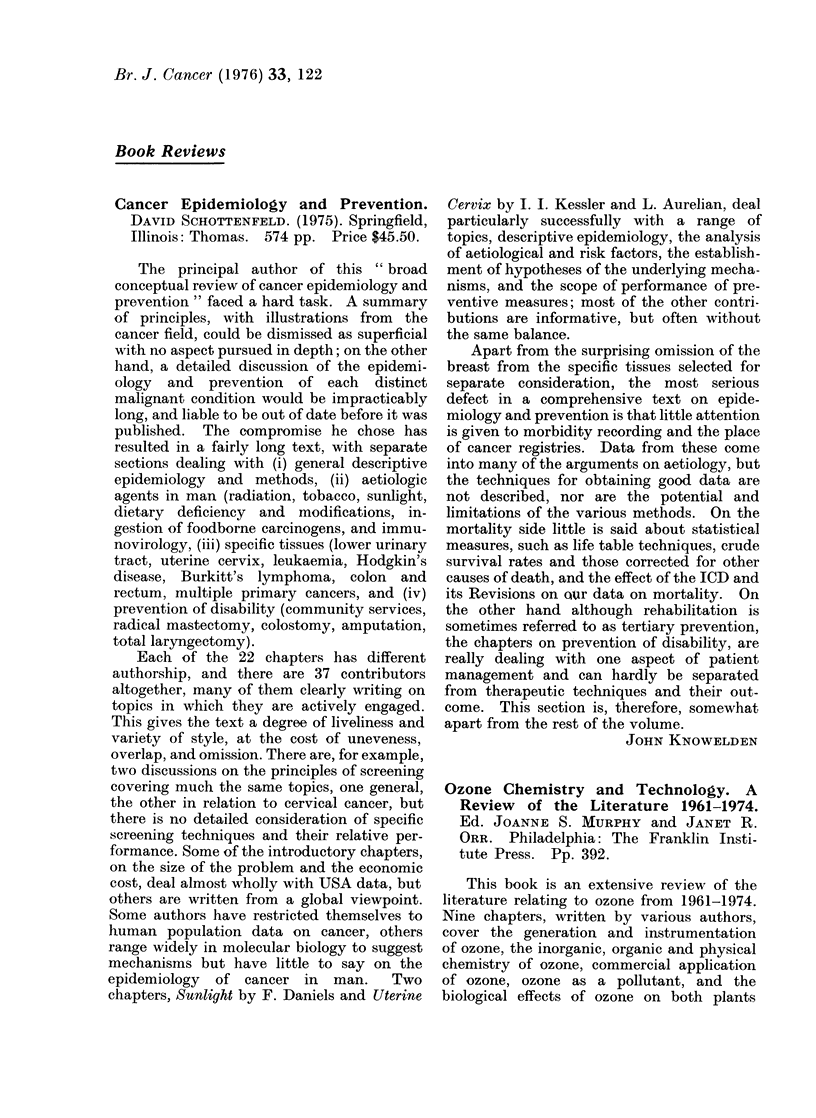# Cancer Epidemiology and Prevention

**Published:** 1976-01

**Authors:** John Knowelden


*Br. J. Cancer* (1976) **33**, 122

### *Book Reviews*

**Cancer Epidemiology and Prevention.** DAVID SCHOTTENFELD. (1975). Springfield, Illinois: Thomas. 574 pp. Price $45.50.

The principal author of this " broad conceptual review of cancer epidemiology and prevention " faced a hard task. A summary of principles, with illustrations from the cancer field, could be dismissed as superficial with no aspect pursued in depth; on the other hand, a detailed discussion of the epidemiology and prevention of each distinct malignant condition would be impracticably long, and liable to be out of date before it was published. The compromise he chose has resulted in a fairly long text, with separate sections dealing with (i) general descriptive epidemiology and methods, (ii) aetiologic agents in man (radiation, tobacco, sunlight, dietary deficiency and modifications, ingestion of foodborne carcinogens, and immunovirology, (iii) specific tissues (lower urinary tract, uterine cervix, leukaemia, Hodgkin's disease, Burkitt's lymphoma, colon and rectum, multiple primary cancers, and (iv) prevention of disability (community services, radical mastectomy, colostomy, amputation, total laryngectomy).

Each of the 22 chapters has different authorship, and there are 37 contributors altogether, many of them clearly writing on topics in which they are actively engaged. This gives the text a degree of liveliness and variety of style, at the cost of uneveness, overlap, and omission. There are, for example, two discussions on the principles of screening covering much the same topics, one general, the other in relation to cervical cancer, but there is no detailed consideration of specific screening techniques and their relative performance. Some of the introductory chapters, on the size of the problem and the economic cost, deal almost wholly with USA data, but others are written from a global viewpoint. Some authors have restricted themselves to human population data on cancer, others range widely in molecular biology to suggest mechanisms but have little to say on the epidemiology of cancer in man. Two chapters, *Sunlight* by F. Daniels and *Uterine Cervix* by I. I. Kessler and L. Aurelian, deal particularly successfully with a range of topics, descriptive epidemiology, the analysis of aetiological and risk factors, the establishment of hypotheses of the underlying mechanisms, and the scope of performance of preventive measures; most of the other contributions are informative, but often without the same balance.

Apart from the surprising omission of the breast from the specific tissues selected for separate consideration, the most serious defect in a comprehensive text on epidemiology and prevention is that little attention is given to morbidity recording and the place of cancer registries. Data from these come into many of the arguments on aetiology, but the techniques for obtaining good data are not described, nor are the potential and limitations of the various methods. On the mortality side little is said about statistical measures, such as life table techniques, crude survival rates and those corrected for other causes of death, and the effect of the ICD and its Revisions on our data on mortality. On the other hand although rehabilitation is sometimes referred to as tertiary prevention, the chapters on prevention of disability, are really dealing with one aspect of patient management and can hardly be separated from therapeutic techniques and their outcome. This section is, therefore, somewhat apart from the rest of the volume.

JOHN KNOWELDEN